# Monotherapy *versus* combination therapy for treatment of carbapenem-resistant *Acinetobacter baumannii* complex infections: results from an observational study

**DOI:** 10.62675/2965-2774.20260352

**Published:** 2026-05-20

**Authors:** Sidnei Umberto Bertholdi, João Paulo Telles, Igor Mochiutti de Melo, Daniela Kallíope de Sá Paraskevopoulos, Carolina Hikari Yamada, Allan Homero dos Santos, Felipe Francisco Tuon, João Silva de Mendonça, Augusto Yamaguti, Thaís Guimarães

**Affiliations:** 1 Department of Infection Control Hospital do Servidor Público Estadual “Francisco Morato de Oliveira” São Paulo SP Brazil Department of Infection Control, Hospital do Servidor Público Estadual “Francisco Morato de Oliveira” - São Paulo (SP), Brazil.; 2 Department of Infection Control Hospital Universitario Evangélico Mackenzie Curitiba PR Brazil Department of Infection Control, Hospital Universitario Evangélico Mackenzie - Curitiba (PR), Brazil.; 3 Escola de Medicina Pontifícia Universidade Católica do Paraná Curitiba PR Brazil Escola de Medicina, Pontifícia Universidade Católica do Paraná - Curitiba (PR), Brazil.

**Keywords:** Cross infection, Monotherapy, Acinetobacter baumannii, Pneumonia, ventilator-associated, Sulbactam, Carbapenems, Intensive care units

## Abstract

**Objective:**

To evaluate the efficacy of monotherapy *versus* combination therapy in the treatment of carbapenem-resistant *Acinetobacter baumannii* infections.

**Methods:**

A retrospective observational study was conducted in two tertiary hospitals in Brazil from 2018 to 2022. Patients diagnosed with bloodstream infections or ventilator-associated pneumonia caused by carbapenem-resistant *Acinetobacter baumannii* were included. Data on demographics, clinical characteristics, antimicrobial regimens, and outcomes were collected. Statistical analyses, including multivariate logistic regression, were performed to identify predictors of mortality.

**Results:**

Among 123 patients (median age: 61 years), 86.2% were treated in the intensive care unit, 28-day mortality was 59,3%, and the overall mortality was 73.2%. Combination therapy was more frequently used (75.6%) and typically involved polymyxin-based regimens (71.5%). Sulbactam-containing regimens were employed in 16.3% of cases. Monotherapy was more common in ventilator-associated pneumonia (33.3%) than in bloodstream infections (14%). Mortality rates were similar between monotherapy and combination therapy (73.3% *versus* 73.1%; p = 0.982). In the multivariable logistic regression model, only the APACHE II score (OR = 1.12; 95%CI 1.05 - 1.21; p = 0.001) remained independently associated with mortality. Sulbactam-based therapy was not independently associated with survival (OR = 0.80; 95%CI 0.22 - 2.85; p = 0.725).

**Conclusion:**

Our findings suggest that neither monotherapy nor combination therapy is associated with lower mortality in this cohort of carbapenem-resistant *Acinetobacter baumannii* infections, likely due to the severity of the baseline illness. Further prospective studies are needed to confirm these results and refine treatment guidelines.

## INTRODUCTION

The carbapenem-resistant *Acinetobacter baumannii* (CRAB) complex is a significant nosocomial pathogen, notable for its ability to cause in-hospital outbreaks and its extensive antimicrobial resistance profile. Currently, no ideal therapeutic options are available for managing infections caused by this organism.^1^Carbapenem-resistant *Acinetobacter baumannii* is particularly problematic in critically ill and immunocompromised patients. These infections are associated with alarmingly high mortality rates of 40 - 60% worldwide, reaching up to 80% in a single cohort.^2,3,4,5^ Carbapenem resistance in *Acinetobacter spp.* is largely driven by Ambler class D beta-lactamases (OXA), especially OXA-23 and OXA-51, which play a key epidemiological role.^6,7^

Therapeutic options for CRAB infections are limited. Available treatments include sulbactam, an older drug with intrinsic activity against *Acinetobacter spp.*, tigecycline, and polymyxins, which remain the cornerstone of treatment due to their rare resistance profile.^6-8^International guidelines also mention cefiderocol as an option of treatment.^1^However, this drug is currently not available in Brazil. Commercially available beta-lactams combined with novel beta-lactamase inhibitors are ineffective against CRAB because they do not inhibit OXA-like enzymes, the primary mechanism of carbapenem resistance in this species.^9^A new β-lactamase inhibitor with action against OXA-type enzymes, durlobactam associated with sulbactam, has been studied and is promising in the treatment of CRAB,^10^but is not yet commercially available in Brazil. Durlobactam/sulbactam has been studied in a phase 3 clinical trial (ATTACK trial), that demonstrated better outcomes compared with colistin-based treatments.^11^

The role of combination therapy remains controversial in CRAB infections. While combination regimens have been explored for carbapenem-resistant *Enterobacterales* in both retrospective and prospective studies, their efficacy against CRAB has not been definitively established. Two prospective studies comparing colistin monotherapy to colistin combined with meropenem or other drugs have failed to demonstrate the superiority of combination therapy.^10,11^ Although these studies analyzed carbapenem-resistant *Gram*-negative bacilli broadly, most included cases were ventilator-associated pneumonia (VAP) caused by CRAB.

Given the high mortality associated with CRAB infections and the lack of a gold-standard treatment, this study aimed to to evaluate the efficacy of monotherapy *versus* combination therapy in the treatment of carbapenem-resistant *A. baumannii* infections.

## METHODS

### Study design

This was a retrospective observational study evaluating the clinical outcomes of monotherapy *versus* combination therapy for infections caused by CRAB. The study adhered to established principles for observational research, including the STROBE statement. The analyses are exploratory and focused on prognostic associations and therapeutic regimens rather than establishing causal relationships. Data were collected from January 1st, 2018, to December 31st, 2022, to ensure a comprehensive assessment of clinical practices during this period.

### Setting

The study was conducted in two tertiary hospitals in Brazil: *Hospital do Servidor Público Estadual “Francisco Morato de Oliveira”* (HSPE), located in São Paulo (SP), and *Hospital Universitario Evangélico Mackenzie*, located in Curitiba (PR). *Hospital do Servidor Público Estadual “Francisco Morato de Oliveira”* is a public teaching hospital with 751 beds, including 51 adult intensive care unit (ICU) beds, 8 pediatric ICU beds, and 12 neonatal ICU beds that serve public servants of the state of São Paulo. *Hospital Universitario Evangélico Mackenzie* is a philanthropic teaching institution with 500 beds, including 61 adult ICU beds, 10 pediatric ICU beds, and 40 neonatal ICU beds. It provides care across various medical and surgical specialties, including kidney and liver transplantation.

Both institutions have in-house microbiology laboratories equipped with the BACTEC™ 9000® system for blood culture processing and the Phoenix BD™ system for antimicrobial susceptibility testing. These systems follow the guidelines and interpretative criteria established by the Brazilian Committee on Antimicrobial Susceptibility Testing (BrCAST). This independent national committee sets standards for microbiological tests and results.^12^Antimicrobial susceptibility testing was interpreted according to BrCAST criteria, which follow the same breakpoints and standards as EUCAST (European Committee on Antimicrobial Susceptibility Testing).

### Participants

Patients were included in the study if they were over 18 years of age, had a confirmed diagnosis of either bloodstream infections (BSI) or VAP caused by CRAB, met the National Healthcare Safety Network (NHSN) criteria for nosocomial infections,^13^and received at least 48 hours of antimicrobial therapy prescribed after CRAB isolation in culture according to attending physician criteria.

Patients were excluded if they were receiving palliative care or if CRAB was isolated from sites other than BSI or VAP. Eligible cases were identified through the infection control databases at both hospitals.

### Data collection and variables

Clinical and laboratory data were extracted from medical records. They included demographic characteristics (age, gender), date of hospital admission and discharge, date of infection diagnosis, ICU admission, Acute Physiology and Chronic Health Evaluation (APACHE II) scores upon ICU admission, Charlson comorbidity index (CCI), use of vasoactive drugs, and comorbidities such as *diabetes mellitus*, chronic obstructive pulmonary disease (COPD), hypertension, malignancies, and liver disease. Microbiological data included the susceptibility profiles of *A. baumannii* isolates. Therapeutic regimens were defined as monotherapy (CRAB treatment with either polymyxin or ampicillin/sulbactam) and combination therapy (two or more antimicrobial agents prescribed with the intention and possibility to treat CRAB) for more than 48 hours. Polymyxin dosing was not adjusted for renal function, and ampicillin/sulbactam was not used at higher doses in all patients receiving this drug. Treatment duration was also recorded. Mortality after 28 days of hospitalization was the primary outcome of interest.

Microbiological evaluations were performed in accordance with BrCAST standards. Antimicrobial susceptibility testing was conducted using Phoenix BD™ for most antimicrobials, while polymyxin susceptibility was assessed using Poli CIMBac®, a microdilution system for determining minimum inhibitory concentrations (MICs). Minimum inhibitory concentration values > 2μg/mL were classified as resistant. Susceptibility to ampicillin/sulbactam was reported based on culture results obtained from antimicrobial susceptibility testing performed and standardized at the time.

### Bias

To minimize bias, data collection was standardized using a predefined checklist to ensure uniformity in patient selection and data extraction. Only microbiologically confirmed cases were included, and palliative care patients (explicitly stated on the records) were excluded to prevent confounding from unrelated mortality causes.

### Study size

The study included 123 patients identified from the infection control databases of both institutions, based on the availability of microbiologically confirmed CRAB cases during the study period.

### Statistical analysis

Data were managed and analyzed using Statistical Package for the Social Sciences (SPSS), version 21 (IBM® Statistics). Descriptive statistics were used to summarize the data, with categorical variables expressed as percentages and continuous variables as medians and interquartile ranges (IQRs). The comparison of continuous variables between groups was performed using the T-test or Mann-Whitney U test, depending on data distribution. Categorical variables were analyzed using chi-square or Fisher’s exact test, as appropriate.

Univariate analyses were performed to identify variables associated with 28-day mortality. Variables with p < 0.05 in univariate testing were entered into a multivariable logistic regression model to assess independent associations with mortality. Results from the multivariable model are presented as odds ratios (OR) with 95% confidence intervals (95%CIs). Missing data were minimized by thorough record review, and cases with incomplete data were excluded from the analysis. No imputation methods were applied for missing data.

### Ethical considerations

This study was conducted in compliance with ethical standards for retrospective research. Institutional review board approval was obtained from both participating hospitals. It is filed in the ethics committee with the number CAAE: 31558020.8.0000.0103. Given its retrospective design and the use of anonymized data, the requirement for informed consent was waived.

## RESULTS

### Participants

A total of 123 patients were analyzed during the study period. All patients met the eligibility criteria, which included a confirmed diagnosis of BSI or VAP caused by CRAB. No patients were excluded due to missing critical data or loss to follow-up, as the study focused solely on hospital outcomes during the admission period.

### Descriptive data

Table 1 summarizes the demographic and clinical characteristics of the cohort and presents antimicrobial susceptibility patterns and treatment-related characteristics. The median age of the cohort was 61 years (IQR = 45 - 72), and 56.9% were male. Most patients (86.2%) had their infections diagnosed in the intensive care unit (ICU). Coronavirus disease 2019 (COVID-19) co-infection was present in 69.1% of the cases. Regarding comorbidities, 31% of patients had diabetes mellitus, 10.6% had chronic kidney disease, and 8.1% had COPD. The median CCI was 3 (IQR = 1 - 4), and the median APACHE II score was 20.5 (IQR = 12.25 - 26). Vasopressor use was documented in 74.8% of patients, underscoring the severity of their clinical status.

**Table 1:** Demographic, clinical, microbiological, and treatment-related characteristics according to antimicrobial regimen

Variables	Total (n = 123)	Monotherapy (n = 31)	Combination therapy (n = 92)	p value
Male gender	70 (56.9)	18 (58)	52 (56 )	0.694
Age	61 (45 - 72)	56 (43 - 62)	63 (48 - 73)	0.048
Length of stay	22 (9.7 - 33.7)	28 (19 - 44)	18 (6 - 32)	0.290
Length of stay after diagnosis	15 (7 - 25.5)	16 (8 - 27)	15 (7 - 24)	0.958
COVID-19	85 (69.1)	26 (83.8)	59 (64)	0.017
Diabetes	38 (31)	5 (16.1)	33 (35)	0.064
Chronic kidney disease	13 (10.6)	2 (6.4)	11 (11.9)	0.424
Previous acute myocardial infarction	7 (5.7)	0	7 (7.6)	0.122
Heart failure	9 (7.3)	2 (6.4)	7 (7.6)	0.875
Neurological disease	9 (7.3)	1 (3.2)	8 (8.6)	0.354
COPD	10 (8.1)	1 (3.2)	9 (9.7)	0.269
Hypertension	63 (51.2)	17 (55)	46 (50)	0.492
Neoplasms	8 (6.5)	2 (6.4)	6 (6.5)	0.967
Cirrhosis	1 (0.8)	0	1 (1)	0.568
Charlson score	3 (1 - 4)	3 (0 - 4.25)	3 (1 - 4.25)	0.278
APACHE II upon ICU admission	20.5 (12.2 - 26)	17 (10 - 25)	21 (13 - 26)	0.469
Infection diagnosed in the ICU	106 (86.2)	26 (86.6)	80 (86)	0.929
Vasopressor use	92 (74.8)	26 (84)	66 (71)	0.085
Other bacterial co-infection with *A. baumannii*	26 (21.1)	4 (13)	22 (23.9)	0.229
Bloodstream infection, n (%)	57 (46.3)	8 (26)	49 (53)	0.013
Ventilator-associated pneumonia	66 (53.7)	22 (71)	44 (47.8)	0.013
Treatment duration		7 (5.25 - 7)	7 (6 - 10)	0.135
Polymyxin-containing regimens	88 (71.5)	26 (83)	62 (67)	0.035
Sulbactam-containing regimens	20 (16.3)	1 (3.2)	19 (20.6)	0.027
28-day mortality	73 (59.3)	18 (58)	55 (59.7)	0.890
Antimicrobial susceptibility				
Meropenem	0	0	0	-
Sulbactam	5 (4)	1 (3.2)	4 (4.3)	0.808
Gentamicin	3 (2.4)	0	3 (3.2)	0.319
Amikacin	7 (5.6)	1 (3.2)	6 (6.5)	0.521
Polymyxin	104 (84.5)	25 (80.6)	79 (85)	0.711

COPD - chronic obstructive pulmonary disease; APACHE II - Acute Physiology and Chronic Health Evaluation II; ICU – intensive care unit. Results expressed as n (%) or median (interquartile range).

### Main results

The therapeutic regimens used for CRAB infections are detailed in table 2. Combination therapy was the most used strategy, employed in 92 patients (75%), while monotherapy was used in 31 patients (25%). Polymyxin-based regimens were predominant (69%), with 41% of patients receiving polymyxin B combined with meropenem.

**Table 2:** Treatments used for carbapenem-resistant *Acinetobacter baumannii* infections

Variables	
Monotherapy	31 (25.2)
Polymyxin-based therapy	85 (69)
Polymyxin B	27 (21.9)
Polymyxin B + meropenem	51 (41.4)
Polymyxin B + imipenem	2 (1.6)
Polymyxin B + amikacin	4 (3.2)
Polymyxin B + gentamicin	1 (0.8)
Sulbactam-based therapy	16 (13)
Ampicillin/sulbactam	1 (0.8)
Ampicillin/sulbactam + tigecycline + ceftazidime/avibactam	1 (0.8)
Ampicillin/sulbactam + meropenem	10 (8.1)
Ampicillin/sulbactam + gentamicin	4 (3.2)
Sulbactam and polymyxin-based therapy	4 (3.2)
Polymyxin B + ampicillin/sulbactam	3 (2.4)
Polymyxin B + meropenem + ampicillin/sulbactam	1 (0.8)
Others	19 (12.2)
Doxycycline	1 (0.8)
Meropenem	2 (1.6)
Meropenem + doxycycline	1 (0.8)
Meropenem + amikacin	2 (1.6)
Meropenem + gentamicin	7 (5.7)
Meropenem + gentamicin + doxycycline	1 (0.8)
Meropenem + doxycycline + amikacin	5 (4.1)
Treatment duration	7 (6 - 10)

Results expressed as n (%) or median (interquartile range).

When comparing monotherapy to combination therapy, patients receiving combination therapy were older (median age: 63 years, IQR = 48 - 73) than those on monotherapy (median age: 56 years, IQR = 43 - 62; p = 0.048) (Table 1). Patients with BSI presented a higher prevalence of combination therapy (52.6% *versus* 26.6%; p = 0.013), while monotherapy was more prevalent in VAP (73% *versus* 47%; p = 0.013). However, there was no significant difference in overall mortality between the two groups (73.1% *versus* 73.3%; p = 0.982).

**Figure 1:**
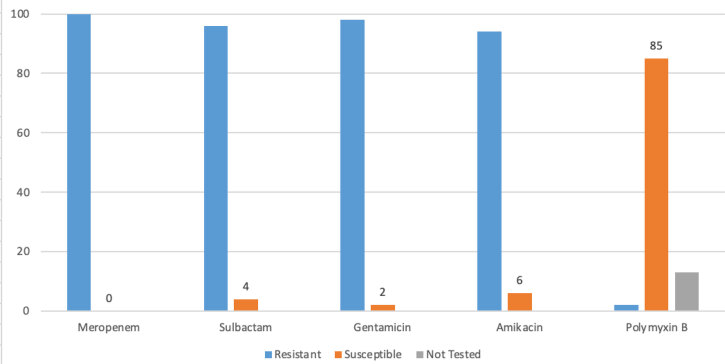
Susceptibility profile of *Acinetobacter baumannii* complex isolates (%) (n = 123).

The susceptibility profile of *A. baumannii* isolates is shown in figure 1. Polymyxins demonstrated favorable susceptibility, with 85% of isolates being sensitive. In contrast, sulbactam and aminoglycosides exhibited poor susceptibility, with only ~5% of isolates being susceptible.

### Outcomes

The 28-day mortality rate was 59.3% (73/123), and the overall mortality was 73.2% (90/123). Mortality rates by clinical characteristics and therapeutic regimens are provided in table 3. In the univariate analysis, patients who died were older (median 65 *versus* 53 years; p = 0.002), had higher Charlson scores (median 3 *versus* 2.5; p = 0.016), and had substantially higher APACHE II scores (median 23.5 *versus* 14; p < 0.001) compared with survivors. Neither monotherapy nor the sulbactam-based regimen was associated with survival.

**Table 3:** Univariate analysis of clinical and epidemiological characteristics regarding 28-day mortality

Variables	Death (n = 73)	Survivor (n = 50)	p value
Male gender	39 (53)	31 (62)	0.346
Age	65 (55 -7 5)	53 (41 - 65)	0.002
HIV	0	0	-
Diabetes	27 (36.9)	12 (24)	0.147
Chronic kidney disease	10 (13)	3 (6)	0.173
Previous acute myocardial infarction	6 (8)	1 (2)	0.144
Heart failure	8 (11)	1 (2)	0.061
Neurological comorbidity	5 (6.8)	4 (8)	0.826
COPD	8 (11)	2 (4)	0.165
Hypertension	38 (52)	25 (50)	0.823
Neoplasms	6 (8.2)	2 (4)	0.351
Cirrhosis	1 (1.3)	0	1
Charlson score	3 (2 - 5)	2.5 (0.75 - 4)	0.016
APACHE II	23.5 (18 - 28.5)	14 (8 - 20)	< 0.001
Infection diagnosed in the ICU	63 (86)	43 (86)	1
Vasopressor use	56 (76)	36 (72)	0.554
Bacterial co-infection with *A. baumannii*	18 (24.6)	8 (16)	0.248
Bloodstream infection	40 (54.7)	17 (34)	0.23
Ventilator-associated pneumonia	33 (45)	33 (66)	0.23
COVID-19	50 (68)	35 (70)	0.859
Antimicrobial within 24 hours after culture	48 (65)	37 (74)	0.217
Monotherapy	18 (24.6)	13 (26)	0.866
Combination therapy	55 (75)	36 (72)	0.680
Regimens with sulbactam therapy	9 (12)	10 (20)	0.248

COPD - chronic obstructive pulmonary disease; APACHE II - Acute Physiology and Chronic Health Evaluation II; ICU – intensive care unit. Results expressed as n (%) or median (interquartile range).

### Multivariate analysis

Two multivariable logistic regression models were constructed to evaluate prognostic associations with 28-day mortality.

In the main model, including monotherapy as the exposure, only APACHE II score remained independently associated with death (OR = 1.12; 95%CI 1.05 - 1.21; p = 0.001), while monotherapy was not a significant factor (OR = 2.01; 95%CI 0.61 - 6.62; p = 0.25). Full model outputs are presented in table 4.

**Table 4:** Multivariable logistic regression assessing the association between monotherapy and 28-day mortality

	Odds ratio (95%CI)	p value
Age	1.01 (0.98 - 1.04)	0.61
Charlson score	1.07 (0.81 - 1.40)	0.65
APACHE II	1.12 (1.05 - 1.21)	0.001
Monotherapy	2.01 (0.61 - 6.62)	0.25

95%CI - 95% confidence interval; APACHE II - Acute Physiology and Chronic Health Evaluation II. All continuous variables were entered as linear predictors: age (per additional year), Charlson comorbidity index (per point), and APACHE II score (per point).

In the second model, focused on sulbactam-based combination therapy, APACHE II again remained the only independent predictor (OR = 1.12; 95% CI 1.05 - 1.21; p = 0.001), and sulbactam-based therapy showed no association with mortality (OR = 0.80; 95% CI 0.22 - 2.85; p = 0.72).

## DISCUSSION

Our study evaluated 123 patients, predominantly male, with a median age of 61 years and a median length of hospital stay of more than 2 weeks. A significant prevalence of COVID-19 was observed in this cohort, affecting 69.2% of the patients. The Severe acute respiratory syndrome coronavirus 2 (SARS-CoV-2) pandemic has markedly influenced the epidemiology of healthcare-associated infections (HAIs) globally, particularly by increasing the incidence of multidrug-resistant organisms, likely driven by higher antimicrobial consumption.^14^A Brazilian retrospective study comparing the pre-pandemic and pandemic periods reported an increased incidence of CRAB-associated HAIs during the pandemic.^15^Similarly, a recent systematic review on the impact of the pandemic on microbial resistance identified three studies documenting a substantial increase in CRAB incidence, ranging from 107.5% to 621%.^16^

In terms of severity, our patients had a median CCI of 3 and an APACHE II score of 20.5, indicating a predicted mortality of 30%. Notably, 86.2% of infections were diagnosed in the ICU, and approximately 21% of patients presented co-infection pathogens, underscoring the critical condition of these patients. The 28-day mortality was 59.3%, and the overall mortality rate was 73.2%, consistent with findings in the literature. A study conducted at *Hospital das Clínicas* of *Universidade de São Paulo* identified *A. baumannii* infections, patient age, and *diabetes mellitus* as factors associated with worse prognosis. The study concluded that *A. baumannii* infections, especially in critically ill patients and those with bacteremia, are associated with lower survival rates compared to infections caused by other pathogens.^3^

Antimicrobial susceptibility testing revealed 84.5% sensitivity to polymyxins, whereas susceptibility to aminoglycosides and sulbactam was approximately 5%. Challenges in susceptibility testing for polymyxins and sulbactam were noted. Both BrCAST and EuCAST currently discourage routine susceptibility testing for sulbactam due to the lack of ecological cut-off points and the complexity of recommended methods.^12^For polymyxins, microdilution in broth is the recommended technique. Surveillance data from 2023 on BSI in adult ICUs in São Paulo reported polymyxin sensitivity at approximately 96% in *Acinetobacter* species.^17^While these in vitro data support polymyxins as a therapeutic option, additional in vivo studies are needed to better assess their efficacy and safety.^18^As previously mentioned, EUCAST and BrCAST do not have standardized methods for susceptibility testing of sulbactam; however, the IDSA guideline recommends its use as part of treatment regimens, often at high doses, even with limited evidence.^19^

During most of the study period, the use of optimized high-dose sulbactam was not part of routine practice in Brazil, as these recommendations were only incorporated into international guidelines after 2022. The number of patients treated with sulbactam in this sample is too small to inform treatment decisions, and prospective studies at higher doses are needed to better evaluate its efficacy. This is one of the limitations of our study and may have influenced the outcomes observed in these patients.

As described in the results, we observed a high 28-day mortality rate. In the univariate analysis, some clinical characteristics were associated with higher mortality (older age, higher Charlson score, and higher APACHE II score upon ICU admission). However, in the multivariate model, only the APACHE II score remained an independent predictor of 28-day mortality. Different antimicrobial treatment regimens (polymyxin monotherapy or sulbactam-based therapy) were also not associated with lower mortality rate. Susceptibility testing of microbial isolates confirmed high polymyxin sensitivity but limited sulbactam activity, although the MIC breakpoints for the latter drug are not standardized.

Combination therapy was the most prevalent treatment strategy in this cohort, used in 75% of patients, with polymyxin-based regimens being the most common (69%). Sulbactam-containing regimens were employed in 16.3% of cases. Two patients were treated with meropenem despite culture results (i.e., one died before changing therapy, and the other presented clinical improvement despite culture results, but died after 17 days due to other complications). When comparing monotherapy to combination therapy, significant differences were observed. Patients receiving combination therapy were older and more likely to have BSI and to receive sulbactam-based regimens. In contrast, monotherapy was more frequently prescribed for VAP and COVID-19 infection, and polymyxin-based therapy was also more common in the monotherapy group. These patterns likely reflect clinical decision-making rather than treatment effect.

Our findings suggest that monotherapy (as previously defined) was not associated with higher mortality compared to combination therapy in this cohort. In this context, the absence of an association between treatment strategies and mortality in the multivariate analysis should be interpreted with caution, as this study was not designed or powered to compare therapeutic regimens.

The Infectious Diseases Society of America (IDSA) recommends sulbactam in combination therapy for moderate-to-severe CRAB infections, regardless of susceptibility results. However, this recommendation is debated, as studies have failed to demonstrate significant impacts on mortality.^20^ In our study, neither monotherapy, co-infection, nor sulbactam-based therapy was significantly associated with higher mortality (Table 3).

In the multivariate logistic regression analysis, only the APACHE II score remained independently associated with 28-day mortality. Other variables that appeared significant in the univariate analysis - including vasopressor use, comorbidities, and antimicrobial regimen - did not retain significance after adjustment. These findings suggest that clinical presentation at the time of diagnosis may be the most important predictor of survival: patients with lower APACHE II scores tended to survive this infection in this cohort. It is not clear why some patients are frankly septic, and others are stable when diagnosed with CRAB infection. However, it is known that infection by CRAB itself is associated with higher mortality, as compared to VAP and BSI caused by other agents.^3^

Overall, the management of CRAB infections remains a major clinical challenge, particularly in the absence of strong evidence to guide monotherapy *versus* combination therapy and limited therapeutic options.

A recent international randomized controlled trial (OVERCOME) compared colistin monotherapy to colistin combined with meropenem for BSI or pneumonia caused by resistant *Gram*-negative bacilli. This study, in which *A. baumannii* accounted for 78% of pathogens, found no significant difference in 28-day mortality (43% *versus* 37%; p = 0.17).^9^Similarly, an open-label study published in 2018 found no advantage of combination therapy over monotherapy, with the addition of meropenem to colistin showing no improvement in outcomes.^21^

These findings, along with ours, suggest that combination therapy may not be necessary for pneumonia caused by carbapenem-resistant *Acinetobacter* spp. However, the small number of BSI cases treated with monotherapy in our study (n = 8) limits the generalizability of these results and warrants cautious interpretation.

The treatment of CRAB infections remains an area of significant clinical uncertainty due to the lack of novel therapeutic agents and the high mortality associated with these infections. There is an urgent need for well-designed, prospective randomized trials to address critical knowledge gaps, particularly regarding the role of monotherapy in BSIs, non-polymyxin-based regimens, and the efficacy of high-dose sulbactam.

The external validity of our findings should be interpreted with caution. Brazil is a continental country, and resistance patterns are considerably different across states. Efforts have been made to establish a national microbiology database, though underreporting remains a challenge. In Brazil, polymyxins remain among the few active agents against CRAB, and their use is widespread, particularly polymyxin B.^22^This scenario contrasts with practices in high-income countries, where antimicrobials such as sulbactam–durlobactam or cefiderocol are available.

This study has several limitations. It is a retrospective analysis conducted at two hospitals, with a limited sample size and heterogeneity in the antimicrobial regimens used in combination therapy, determined by attending physicians’ judgment. The study did not evaluate antimicrobial dosing, potential adverse effects, or renal function in patients. Resistance mechanisms were also not analyzed using genotypic methods. Data on the beta-lactam infusion strategy was not available. Although multivariate analysis adjusted for key variables, residual confounding cannot be ruled out. More sophisticated methods, such as propensity score weighting or causal inference approaches, could better address these imbalances in future studies. The relatively small sample size limited the power to detect differences between treatment groups. Additionally, the methodology for sulbactam susceptibility testing, based on automated MIC determination, is not the recommended standard.

Although limited by its retrospective design and sample size, this study contributes to the existing evidence describing outcomes of CRAB infections in real-world clinical practice. Our findings are consistent with prior research showing no significant differences in mortality between monotherapy and combination therapy for CRAB infections. Notably, the statistical power of our analysis was greatest in the VAP subgroup, which may explain why potential differences in other subgroups could not be detected.

## CONCLUSION

In this retrospective cohort, mortality among patients with carbapenem-resistant *A. baumannii* infections remains high and was primarily driven by baseline severity rather than treatment strategy. Neither monotherapy nor combination therapy demonstrated clear survival benefits in this cohort. These results highlight the need for new therapeutic options and prospective trials in settings where durlobactam-sulbactam is not yet available, such as resource-limited settings.
